# Standalone Middle Meningeal Artery Embolization Versus Surgical Evacuation for Nontraumatic Subdural Hematoma: A Propensity-Matched Multicenter Real-World Study

**DOI:** 10.7759/cureus.117205

**Published:** 2026-09-30

**Authors:** Khaled M. S. Alqedra, Ikrima Ahmed, Mohammad Albasuni, Fuad Mohammad Hamdan, Qusai Banihani, Yamen Albawaleez, Yaman Abuobead, Abhishek Prasad

**Affiliations:** 1 Internal Medicine, Faculty of Medicine and Natural Sciences, School of Medicine, Ilia State University, Tbilisi, GEO; 2 Anesthesiology, University of Texas Health Medical Center, Houston, USA

**Keywords:** ischemic stroke, middle meningeal artery embolization, mortality, propensity score matching, retrospective cohort study, seizure, subdural hematoma, surgical evacuation, trinetx

## Abstract

Objective: The aim of the study was to compare real-world outcomes after standalone middle meningeal artery embolization (MMAE) versus surgical evacuation among adults with inpatient International Classification of Diseases, Tenth Revision, Clinical Modification (ICD-10-CM)-coded nontraumatic subdural hematoma.

Methods: We conducted a retrospective propensity-matched cohort study using TriNetX Global Collaborative Network reports. Adults aged 18 years or older with inpatient ICD-10-CM-coded nontraumatic subdural hemorrhage were assigned to standalone MMAE or surgical evacuation if the qualifying procedure occurred within one month of diagnosis. Patients were matched 1:1 on demographic, cardiovascular, cerebrovascular, renal, hepatic, metabolic, substance use, medication, and laboratory variables. For outcome analyses, the qualifying intervention served as the analytic index event, with follow-up beginning on day 1 after the procedure. Outcomes were all-cause mortality, incident seizure disorder, and ischemic stroke assessed through 3, 6, and 12 months after the qualifying intervention.

Results: After matching, 2,156 patients were included in each cohort. All-cause mortality did not differ significantly at 3 months (245/2,156 (11.4%) vs 283/2,156 (13.1%); risk difference (RD), -1.8%; p=0.08), 6 months (308/2,156 (14.3%) vs 327/2,156 (15.2%); RD, -0.9%; p=0.41), or 12 months (354/2,156 (16.4%) vs 377/2,156 (17.5%); RD, -1.1%; p=0.35). Incident seizures were less frequent after MMAE at 3 months (63/1,888 (3.3%) vs 106/1,798 (5.9%)), 6 months (79/1,888 (4.2%) vs 136/1,798 (7.6%)), and 12 months (97/1,888 (5.1%) vs 157/1,798 (8.7%); all p<0.001); however, this association was not statistically significant in the age >65 years sensitivity analysis. Ischemic stroke risk did not differ significantly at 3 months (90/1,692 (5.3%) vs 81/1,731 (4.7%)), 6 months (103/1,692 (6.1%) vs 97/1,731 (5.6%)), or 12 months (115/1,692 (6.8%) vs 111/1,731 (6.4%)).

Conclusions: In this propensity-matched real-world cohort, standalone MMAE was associated with similar mortality and ischemic stroke risk compared with surgical evacuation. Lower incident seizure risk was observed after MMAE in the primary analysis, but this association was not preserved in the age >65 years sensitivity analysis and should therefore be considered hypothesis-generating. These findings support prospective evaluation in selected patients and should not be interpreted as evidence that MMAE replaces urgent surgical decompression.

## Introduction

Chronic and subacute subdural hematomas are common neurosurgical conditions, particularly among older adults and patients with cerebral atrophy, antithrombotic exposure, or medical frailty. Their clinical presentation is heterogeneous, ranging from mild cognitive or gait disturbance to focal neurologic deficits and depressed consciousness [1]. Subdural hematomas classified as nontraumatic represent a clinically heterogeneous group and may occur in association with factors such as coagulopathy, vascular abnormalities, intracranial hypotension, or neoplastic disease. Importantly, particularly in older adults, minor or unrecognized preceding trauma may not always be documented, making etiologic classification based on diagnostic coding imperfect. As the population ages, the burden of chronic and subacute subdural hematoma is expected to increase, making treatment strategies that reduce morbidity, recurrence, and health care utilization increasingly important [2,3].

Surgical evacuation remains the traditional standard of care for symptomatic subdural hematomas requiring rapid decompression. Burr-hole drainage, craniotomy, and related evacuation techniques can provide immediate relief of mass effect, but they do not directly address the inflammatory and angiogenic membrane biology that contributes to hematoma persistence and recurrence [4,5]. Recurrence after surgical treatment remains clinically relevant, and surgical management may be associated with perioperative complications, readmission, and prolonged recovery in older or medically complex patients [4-7].

Middle meningeal artery embolization (MMAE) has emerged as a minimally invasive treatment strategy based on the vascular contribution of the middle meningeal artery to the fragile neomembranes implicated in nonacute subdural hematoma pathophysiology [2,6,8]. Recent randomized and observational studies have strengthened the evidence for MMAE, particularly as an adjunctive therapy and as a potential standalone option in selected patients [9,10]. However, standalone MMAE and surgical evacuation are not interchangeable in all clinical scenarios: surgery remains essential when urgent decompression is needed, whereas standalone MMAE may be considered in noncritical patients, patients at higher surgical risk, or patients in whom gradual hematoma stabilization is clinically acceptable [11].

Despite growing interest in standalone MMAE, comparative real-world data against surgical evacuation remain limited, and available studies vary in patient selection, outcome definitions, and follow-up. In particular, large multicenter data are needed to better characterize short- and long-term mortality and neurologic outcomes after standalone MMAE compared with surgical evacuation. We focused on the International Classification of Diseases, Tenth Revision, Clinical Modification (ICD-10-CM) nontraumatic category to define a reproducible database cohort without deliberately combining cases explicitly coded as traumatic while recognizing that diagnostic coding cannot exclude undocumented minor trauma. Therefore, we used the TriNetX Global Collaborative Network to compare short- and long-term outcomes among adults with inpatient ICD-10-CM-coded nontraumatic subdural hematoma treated with standalone MMAE or surgical evacuation.

## Materials and methods

Study design and data source

We conducted a retrospective cohort study using the TriNetX Global Collaborative Network (TriNetX LLC, Cambridge, MA), which integrates de-identified electronic health records from 173 healthcare organizations. Outcomes were analyzed after propensity score matching. Because the analysis used de-identified aggregate TriNetX outputs, no patient-level data were accessed for this manuscript. All data are standardized using common ontologies such as ICD-10-CM, Current Procedural Terminology, and RxNorm vocabularies.

Cohort definitions

We identified adults (≥18 years) with inpatient nontraumatic subdural hematoma using ICD-10-CM codes for nontraumatic subdural hemorrhage. For this database study, the term “nontraumatic” reflects the ICD-10-CM classification used for cohort identification rather than adjudicated clinical confirmation of the absence of preceding trauma. Because the TriNetX dataset does not provide sufficiently granular information regarding minor or unrecognized preceding trauma, some misclassification between coded nontraumatic and trauma-associated subdural hematoma may be possible. Treatment groups were assigned using ICD-10-PCS procedure codes, requiring the qualifying intervention to occur within one month of the index subdural hematoma diagnosis to ensure linkage. Intervention cohorts were mutually exclusive for intervention (Table 1).

**Table 1:** Coded concepts and variables used for cohort definition, treatment assignment, and propensity score matching ICD-10-CM, International Classification of Diseases, Tenth Revision, Clinical Modification; MMAE, middle meningeal artery embolization; SDH, subdural hematoma.

Domain	Use in analysis	Code system	Code	TriNetX term/description	Notes
Cohort diagnosis	MMAE and surgery cohort inclusion	ICD-10-CM	I62.03	Nontraumatic chronic subdural hemorrhage	Required with inpatient encounter; included in Group 1A
Cohort diagnosis	MMAE and surgery cohort inclusion	ICD-10-CM	I62.02	Nontraumatic subacute subdural hemorrhage	Required with inpatient encounter; included in Group 1A
Cohort diagnosis	MMAE and surgery cohort inclusion	ICD-10-CM	I62.00	Nontraumatic subdural hemorrhage, unspecified	Included in TriNetX cohort definition
MMAE exposure	MMAE inclusion; surgery exclusion	ICD-10-PCS	03LG3DZ	Occlusion of intracranial artery with intraluminal device, percutaneous approach	Qualifying MMAE procedure within one month after SDH diagnosis
Surgical evacuation	Surgery inclusion; MMAE exclusion	ICD-10-PCS	00C43ZZ	Extirpation of matter from intracranial subdural space, percutaneous approach	Surgical evacuation code within one month after SDH diagnosis
Surgical evacuation	Surgery inclusion; MMAE exclusion	ICD-10-PCS	00C40ZZ	Extirpation of matter from intracranial subdural space, open approach	Surgical evacuation code within one month after SDH diagnosis
Surgical evacuation	Surgery inclusion; MMAE exclusion	ICD-10-PCS	00C44ZZ	Extirpation of matter from intracranial subdural space, percutaneous endoscopic approach	Surgical evacuation code within one month after SDH diagnosis
Surgical evacuation	Surgery inclusion; MMAE exclusion	ICD-10-PCS	009400Z	Drainage of intracranial subdural space with drainage device, open approach	Surgical drainage code within one month after SDH diagnosis
Surgical evacuation	Surgery inclusion; MMAE exclusion	ICD-10-PCS	009430Z	Drainage of intracranial subdural space with drainage device, percutaneous approach	Surgical drainage code within one month after SDH diagnosis
Surgical evacuation	Surgery inclusion; MMAE exclusion	ICD-10-PCS	009440Z	Drainage of intracranial subdural space with drainage device, percutaneous endoscopic approach	Surgical drainage code within one month after SDH diagnosis
Surgical evacuation	Surgery inclusion; MMAE exclusion	ICD-10-PCS	00940ZZ	Drainage of intracranial subdural space, open approach	Surgical drainage code within one month after SDH diagnosis
Surgical evacuation	Surgery inclusion; MMAE exclusion	ICD-10-PCS	00943ZZ	Drainage of intracranial subdural space, percutaneous approach	Surgical drainage code within one month after SDH diagnosis
Surgical evacuation	Surgery inclusion; MMAE exclusion	ICD-10-PCS	00944ZZ	Drainage of intracranial subdural space, percutaneous endoscopic approach	Surgical drainage code within one month after SDH diagnosis
Matching covariate	Propensity score matching	ICD-10-CM range	I10-I1A	Hypertensive diseases	Baseline covariate from TriNetX baseline patient characteristics
Matching covariate	Propensity score matching	ICD-10-CM range	I20-I25	Ischemic heart diseases	Baseline covariate from TriNetX baseline patient characteristics
Matching covariate	Propensity score matching	ICD-10-CM	E11	Type 2 diabetes mellitus	Baseline covariate from TriNetX baseline patient characteristics
Matching covariate	Propensity score matching	ICD-10-CM	I48	Atrial fibrillation and flutter	Baseline covariate from TriNetX baseline patient characteristics
Matching covariate	Propensity score matching	ICD-10-CM	I50	Heart failure	Baseline covariate from TriNetX baseline patient characteristics
Matching covariate	Propensity score matching	ICD-10-CM	N18	Chronic kidney disease	Baseline covariate from TriNetX baseline patient characteristics
Matching covariate	Matching/prior stroke exclusion proxy	ICD-10-CM	I63	Cerebral infarction	Also used to exclude prior outcome in stroke analyses
Matching covariate	Propensity score matching	ICD-10-CM	F17	Nicotine dependence	Baseline covariate from TriNetX baseline patient characteristics
Matching covariate	Propensity score matching	ICD-10-CM range	K70-K77	Diseases of liver	Baseline covariate from TriNetX baseline patient characteristics
Matching covariate	Propensity score matching	ICD-10-CM	F10	Alcohol-related disorders	Baseline covariate from TriNetX baseline patient characteristics
Matching covariate	Propensity score matching	ICD-10-CM	I69	Sequelae of cerebrovascular disease	Baseline covariate from TriNetX baseline patient characteristics
Demographic variable	Age at index	TriNetX	AI	Age at index	Included in matching; not an ICD-10 code
Demographic variable	Sex	TriNetX	M/F	Male/female	Included in matching; not an ICD-10 code
Demographic variable	Race/ethnicity	HL7/CDC code	2106-3	White	Included in matching; not an ICD-10 code
Demographic variable	Race/ethnicity	HL7/CDC code	2054-5	Black or African American	Included in matching; not an ICD-10 code
Demographic variable	Race/ethnicity	HL7/CDC code	2186-5	Not Hispanic or Latino	Included in matching; not an ICD-10 code
Medication covariate	Propensity score matching	ATC	C10	Lipid-modifying agents	Medication class, not ICD-10
Medication covariate	Propensity score matching	ATC	C02	Antihypertensives	Medication class, not ICD-10
Medication covariate	Propensity score matching	ATC	B01AC	Platelet aggregation inhibitors excluding heparin	Medication class, not ICD-10
Medication covariate	Propensity score matching	ATC	B01AF	Direct factor Xa inhibitors	Medication class, not ICD-10
Laboratory covariate	Propensity score matching	TriNetX lab concept	9020	Platelets (#/volume) in blood	Value and measurement-availability balance exported
Laboratory covariate	Propensity score matching	TriNetX lab concept	9024	Creatinine (mass/volume) in serum, plasma, or blood	Value and measurement-availability balance exported
Laboratory covariate	Propensity score matching	TriNetX measurement concept	9083	BMI	Value and measurement-availability balance exported

Table 1 summarizes the coded concepts used for cohort identification, treatment assignment, and propensity-score matching. The “Domain” column indicates the role of each variable in the analysis: cohort diagnosis codes identified eligible patients; MMAE and surgical evacuation procedure codes defined treatment exposure; and demographic, clinical, medication, and laboratory variables represented baseline covariates entered into the propensity-score model. Clinical comorbidities such as hypertension, type 2 diabetes mellitus, atrial fibrillation or flutter, heart failure, chronic kidney disease, and prior cerebrovascular disease were included as potential baseline confounders rather than as separate study cohorts.

Application of these diagnosis, inpatient encounter, procedure, timing, and mutual exclusivity criteria generated the initial unmatched cohorts of 2,276 patients treated with standalone MMAE and 19,973 patients treated with surgical evacuation.

Index event and follow-up

The qualifying subdural hematoma diagnosis was used for cohort identification, and the qualifying MMAE or surgical evacuation procedure was required to occur within one month of that diagnosis. For outcome analyses, the qualifying intervention served as the analytic index event (time zero). Follow-up began on postoperative day 1 (day 1 after the qualifying intervention), and outcomes were assessed from day 1 through 90, 180, and 365 days after the qualifying procedure, corresponding to the 3-, 6-, and 12-month outcome windows, respectively. Thus, outcome follow-up did not begin before treatment. Patients whose qualifying index event occurred 20 years or more before the analysis were excluded by TriNetX; this excluded 10 MMAE patients and 30 surgical patients.

Covariates and propensity score matching

TriNetX performed 1:1 propensity score matching (Figure 1). The matched variables included age, sex, race/ethnicity, hypertension, ischemic heart disease, type 2 diabetes mellitus, atrial fibrillation/flutter, heart failure, chronic kidney disease, prior cerebral infarction, nicotine dependence, liver disease, alcohol-related disorders, prior history of cerebrovascular disease, lipid-modifying agents, antihypertensives, platelet aggregation inhibitors, direct factor Xa inhibitors, platelet count, creatinine, and body mass index. Balance was assessed using standardized differences.

**Figure 1:**
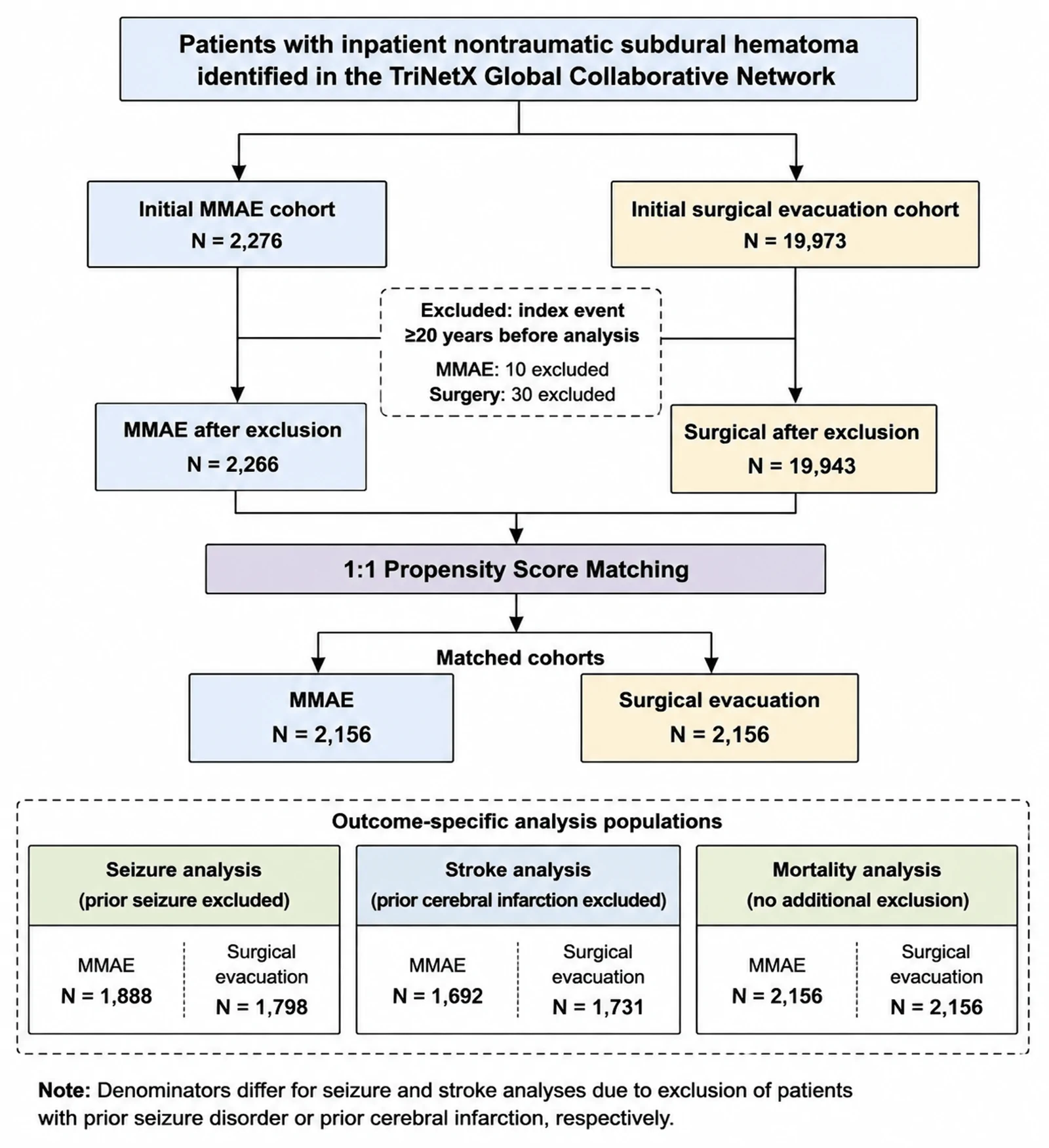
Participant flow and propensity score matching Flow diagram showing identification of patients with inpatient nontraumatic subdural hematoma, formation of the standalone MMAE and surgical evacuation cohorts, exclusion of patients whose index event occurred ≥20 years before analysis, and 1:1 propensity score matching. The matched cohorts included 2,156 patients in each treatment group. Outcome-specific analysis populations were smaller for seizure and ischemic stroke analyses because patients with prior seizure disorders and prior cerebral infarction, respectively, were excluded. Mortality analyses included the full matched cohorts. MMAE, middle meningeal artery embolization.

Outcomes and statistical analysis

The prespecified outcomes available in the three TriNetX reports were all-cause mortality, incident seizures, and ischemic stroke. Patients were excluded if they had the outcome before the outcome window. Outcome-specific denominators therefore differed because patients with prior seizure disorders and prior cerebral infarction were excluded from the incident seizure and ischemic stroke analyses, respectively. Outcomes were analyzed within TriNetX Advanced Analytics using risks with 95% CIs, risk differences, risk ratios, odds ratios, and time-to-event analyses using Kaplan-Meier curves, log-rank testing, and Cox proportional hazards models. Proportional hazards results were interpreted cautiously when proportionality testing was significant. Confounding was minimized with 1:1 nearest neighbor propensity score matching (caliper 0.1) using a logistic model, including age, sex, race/ethnicity, and major comorbidities (Table 2).

**Table 2:** Selected baseline characteristics after propensity score matching BMI, body mass index; MMAE, middle meningeal artery embolization; SD, standard deviation.

Characteristic	MMAE (n=2,156)	Surgery (n=2,156)	p-value	Std diff.
Age at index, mean ± SD	69.3 ± 15.1	69.0 ± 14.7	0.536	0.019
Male sex	1,316 (61.0%)	1,255 (58.2%)	0.058	0.058
Female sex	840 (39.0%)	901 (41.8%)	0.058	0.058
White race	1,335 (61.9%)	1,377 (63.9%)	0.186	0.04
Black or African American race	259 (12.0%)	267 (12.4%)	0.71	0.011
Not Hispanic or Latino	1,598 (74.1%)	1,648 (76.4%)	0.078	0.054
Hypertensive diseases	1,337 (62.0%)	1,303 (60.4%)	0.288	0.032
Ischemic heart diseases	522 (24.2%)	489 (22.7%)	0.236	0.036
Type 2 diabetes mellitus	511 (23.7%)	493 (22.9%)	0.517	0.02
Atrial fibrillation/flutter	420 (19.5%)	398 (18.5%)	0.393	0.026
Heart failure	317 (14.7%)	307 (14.2%)	0.665	0.013
Chronic kidney disease	311 (14.4%)	311 (14.4%)	1	0
Prior cerebral infarction	251 (11.6%)	239 (11.1%)	0.565	0.018
Nicotine dependence	229 (10.6%)	215 (10.0%)	0.483	0.021
Diseases of liver	155 (7.2%)	166 (7.7%)	0.523	0.019
Alcohol-related disorders	146 (6.8%)	139 (6.4%)	0.668	0.013
Sequelae of cerebrovascular disease	120 (5.6%)	120 (5.6%)	1	0
Platelet aggregation inhibitors excluding heparin	264 (12.2%)	256 (11.9%)	0.708	0.011
Direct factor Xa inhibitors	76 (3.5%)	70 (3.2%)	0.613	0.015
Creatinine, mean ± SD, mg/dL	1.2 ± 4.3	1.5 ± 6.9	0.127	0.053
Platelets, mean ± SD, x10^3^/uL	222.9 ± 102.0	230.3 ± 105.3	0.041	0.071
BMI, mean ± SD, kg/m^2^	26.6 ± 6.0	26.8 ± 5.9	0.238	0.043

As a sensitivity analysis, we repeated the matched comparison after excluding patients with structured code proxies for severe or critical presentation, including coma, shock or hemodynamic instability, cardiac arrest, mechanical ventilation, acute respiratory failure, and vasopressor requirement as a surrogate for circulatory shock. An additional sensitivity analysis was performed by restricting the cohort to patients older than 65 years. These sensitivity analyses were intended to evaluate whether the primary findings were robust in a less critically ill inpatient subgroup and in older adults, respectively.

## Results

Cohort assembly and matching

The source query identified 2,276 patients in the MMAE cohort and 19,973 patients in the surgical cohort. After 1:1 matching, 2,156 patients remained in each cohort. Baseline characteristics were generally balanced after matching, with standardized differences below 0.10 for all visible covariates (Table 2, Figure 2)

**Figure 2:**
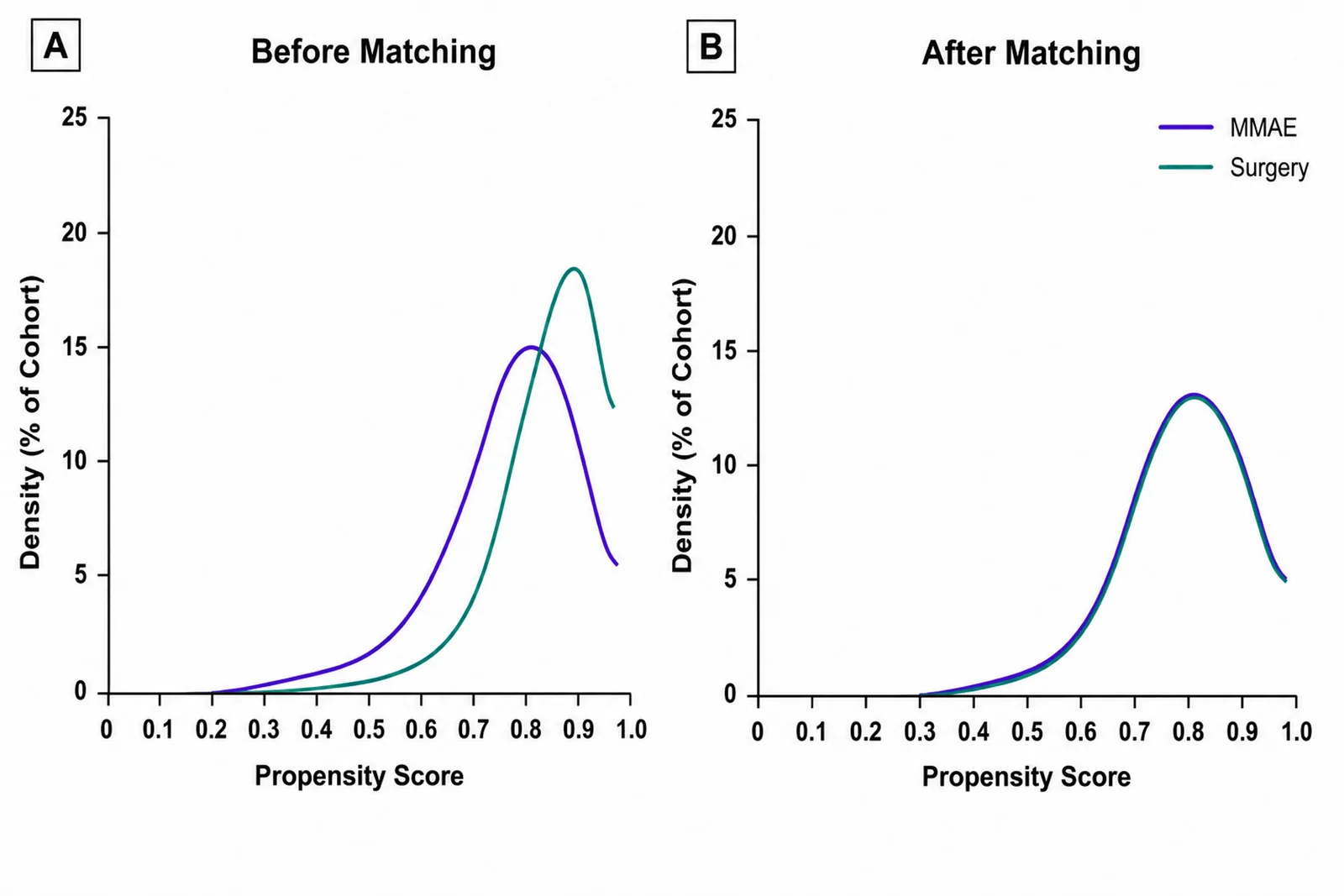
Propensity score density distributions before and after matching (A) Before matching, the standalone middle meningeal artery embolization (MMAE) and surgical evacuation cohorts showed partially overlapping but imbalanced propensity score distributions. (B) After 1:1 propensity score matching, the distributions showed close overlap, indicating improved covariate balance between the treatment groups.

All-cause mortality

All-cause mortality was not significantly different between groups at any follow-up point. At three months, mortality occurred in 245 of 2,156 patients treated with standalone MMAE and 283 of 2,156 patients treated with surgical evacuation (245/2,156 (11.4%) vs 283/2,156 (13.1%); risk difference, -1.8%; 95% CI, -3.7% to 0.2%; p=0.08). Kaplan-Meier survival estimates, which are model-based probability estimates rather than simple proportions, were also similar at three months (87.77% vs 86.31%; log-rank p=0.11).

This pattern remained consistent at later follow-up. At six months, mortality occurred in 308 of 2,156 MMAE patients and 327 of 2,156 surgical patients (308/2,156 (14.3%) vs 327/2,156 (15.2%); risk difference, -0.9%; 95% CI, -3.0% to 1.2%; p=0.41), with nearly identical Kaplan-Meier survival estimates (83.86% vs 83.86%; log-rank p=0.62). At one year, mortality occurred in 354 of 2,156 MMAE patients and 377 of 2,156 surgical patients (354/2,156 (16.4%) vs 377/2,156 (17.5%); risk difference, -1.1%; 95% CI, -3.3% to 1.2%; p=0.35), and Kaplan-Meier survival remained similar between groups (80.45% vs 80.83%; log-rank p=0.80).

Incident seizures

Patients with prior seizure diagnoses were excluded from seizure analyses. At three months, incident seizures occurred in 63 of 1,888 MMAE patients (3.3%) and 106 of 1,798 surgical patients (5.9%; risk difference, -2.6%; 95% CI, -3.9% to -1.2%; p<0.001). At six months, incident seizures occurred in 79 of 1,888 MMAE patients (4.2%) and 136 of 1,798 surgical patients (7.6%; risk difference, -3.4%; 95% CI, -4.9% to -1.9%; p<0.001). At 12 months, incident seizures occurred in 97 of 1,888 MMAE patients (5.1%) and 157 of 1,798 surgical patients (8.7%; risk difference, -3.6%; 95% CI, -5.2% to -2.0%; p<0.001). Kaplan-Meier analyses were directionally consistent at all time points.

Stroke

Patients with prior cerebral infarction were excluded from stroke analyses. Stroke risk did not differ significantly between groups at 3 months (90/1,692 (5.3%) vs 81/1,731 (4.7%); risk difference, 0.6%; 95% CI, -0.8% to 2.1%; p=0.39), 6 months (103/1,692 (6.1%) vs 97/1,731 (5.6%); risk difference, 0.5%; 95% CI, -1.1% to 2.1%; p=0.55), or 12 months (115/1,692 (6.8%) vs 111/1,731 (6.4%); risk difference, 0.4%; 95% CI, -1.3% to 2.0%; p=0.65). Kaplan-Meier log-rank tests were also nonsignificant at all time points (Figure 3; Tables 3, 4).

**Figure 3:**
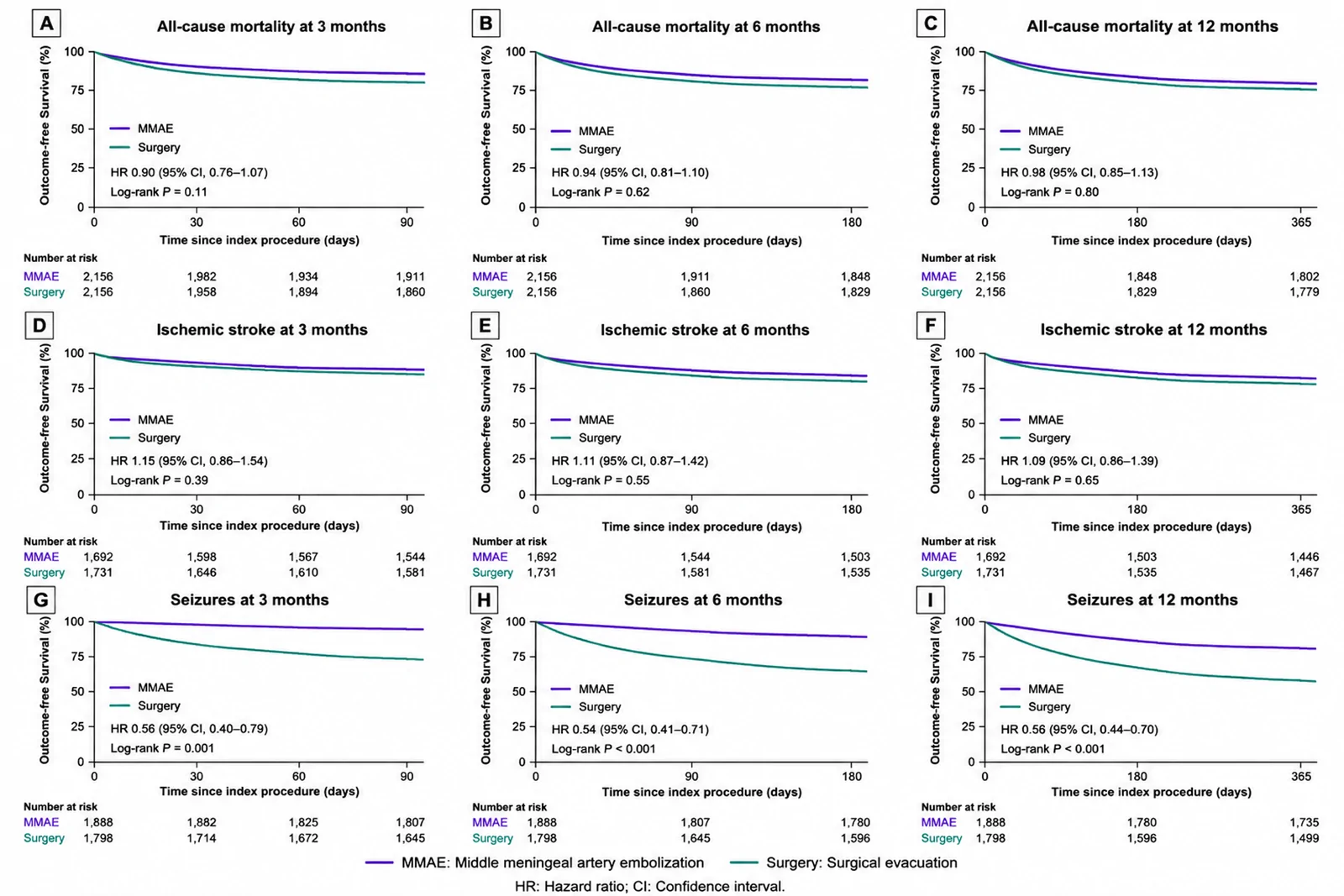
Kaplan-Meier outcome-free survival curves after propensity score matching (A-C) All-cause mortality at 3, 6, and 12 months. (D-F) Ischemic stroke at 3, 6, and 12 months. (G-I) Seizures at 3, 6, and 12 months.

**Table 3:** Risk analysis outcomes after propensity score matching Seizure and stroke analyses excluded patients with the outcome before the time window. CI, confidence interval; MMAE, middle meningeal artery embolization.

Outcome	Time window	MMAE	Surgery	Risk difference (95% CI)	Risk ratio (95% CI)	p-value
All-cause mortality	3 months	245/2,156 (11.4%)	283/2,156 (13.1%)	-1.8% (-3.7 to 0.2)	0.866 (0.738-1.016)	0.078
All-cause mortality	6 months	308/2,156 (14.3%)	327/2,156 (15.2%)	-0.9% (-3.0 to 1.2)	0.942 (0.816-1.087)	0.414
All-cause mortality	12 months	354/2,156 (16.4%)	377/2,156 (17.5%)	-1.1% (-3.3 to 1.2)	0.939 (0.823-1.072)	0.351
Incident seizures	3 months	63/1,888 (3.3%)	106/1,798 (5.9%)	-2.6% (-3.9 to -1.2)	0.566 (0.417-0.768)	<0.001
Incident seizures	6 months	79/1,888 (4.2%)	136/1,798 (7.6%)	-3.4% (-4.9 to -1.9)	0.553 (0.422-0.724)	<0.001
Incident seizures	12 months	97/1,888 (5.1%)	157/1,798 (8.7%)	-3.6% (-5.2 to -2.0)	0.588 (0.461-0.752)	<0.001
Stroke	3 months	90/1,692 (5.3%)	81/1,731 (4.7%)	0.6% (-0.8 to 2.1)	1.137 (0.848-1.523)	0.39
Stroke	6 months	103/1,692 (6.1%)	97/1,731 (5.6%)	0.5% (-1.1 to 2.1)	1.086 (0.830-1.422)	0.546
Stroke	12 months	115/1,692 (6.8%)	111/1,731 (6.4%)	0.4% (-1.3 to 2.0)	1.060 (0.824-1.364)	0.651

**Table 4:** Kaplan-Meier survival analysis summaries The mortality proportionality test was significant at all three time points, so mortality hazard ratios should be interpreted cautiously. Probabilities shown are Kaplan-Meier model-based estimates rather than simple proportions and are therefore not paired with a raw N; corresponding raw event counts and cohort denominators are reported in Table 3. CI, confidence interval; MMAE, middle meningeal artery embolization; PH, proportional hazard.

Outcome	Time window	MMAE end-window probability	Surgery end-window probability	Hazard ratio (95% CI)	Log-rank p	PH test p
All-cause mortality	3 months	Survival 87.77%	Survival 86.31%	0.870 (0.733-1.032)	0.109	0.048
All-cause mortality	6 months	Survival 83.86%	Survival 83.86%	0.961 (0.823-1.123)	0.622	<0.001
All-cause mortality	12 months	Survival 80.45%	Survival 80.83%	0.982 (0.849-1.135)	0.805	0.015
Incident seizures	3 months	Seizure-free 96.31%	Seizure-free 93.49%	0.568 (0.416-0.775)	<0.001	0.178
Incident seizures	6 months	Seizure-free 95.06%	Seizure-free 91.25%	0.562 (0.426-0.742)	<0.001	0.529
Incident seizures	12 months	Seizure-free 93.35%	Seizure-free 89.49%	0.611 (0.474-0.787)	<0.001	0.677
Stroke	3 months	Stroke-free 94.17%	Stroke-free 94.88%	1.158 (0.858-1.563)	0.338	0.329
Stroke	6 months	Stroke-free 93.05%	Stroke-free 93.64%	1.121 (0.850-1.479)	0.419	0.248
Stroke	12 months	Stroke-free 91.75%	Stroke-free 92.44%	1.117 (0.861-1.450)	0.405	0.476

In sensitivity analyses excluding patients with structured code proxies for critical illness, all-cause mortality remained similar between standalone MMAE and surgical evacuation at 3, 6, and 12 months, consistent with the primary analysis. In the age-restricted cohort of patients older than 65 years, mortality also remained similar across all follow-up windows. However, the lower incident seizure risk observed with MMAE in the primary cohort was no longer statistically significant in the age >65 sensitivity analysis.

## Discussion

In this large propensity-matched analysis of adults with ICD-10-CM-coded nontraumatic subdural hematoma, standalone MMAE and surgical evacuation were associated with similar all-cause mortality at 3, 6, and 12 months. Coded ischemic stroke incidence also did not differ significantly between treatment strategies at any follow-up interval. Incident seizure diagnoses were less frequent after MMAE in the primary analysis; however, this association was not preserved in the older adult sensitivity analysis and should therefore be considered hypothesis-generating. Accordingly, the findings do not establish a seizure-preventive effect of MMAE or equivalence between MMAE and surgical evacuation but instead provide observational comparative evidence that warrants prospective validation.

The evidence base for MMAE has expanded rapidly. Recent randomized trials and meta-analyses have shown that MMAE, most often as an adjunct to standard care or surgical evacuation, can reduce hematoma recurrence, progression, or treatment failure without an obvious increase in major neurologic complications [12,13]. These studies support the biologic premise of MMAE: chronic subdural hematoma is not only a mass lesion but also an inflammatory and angiogenic membrane-driven disease, with fragile neovasculature supplied in part by the middle meningeal artery [6]. Embolization targets that vascular supply, whereas surgery provides immediate decompression but does not directly treat the membrane vascularity that may contribute to recurrence [4,9,11,14].

A key limitation of much of the existing randomized evidence is that it does not fully answer whether standalone MMAE can be compared with surgical evacuation as the primary treatment strategy. Adjunctive MMAE trials address whether embolization improves outcomes when added to usual care or surgery, but they do not establish interchangeability between embolization alone and operative evacuation [13,15,16]. Recent observational studies and meta-analyses have begun to address this gap by comparing standalone MMAE with surgery or with medical management, but treatment selection remains highly dependent on patient acuity, radiographic mass effect, symptoms, and institutional practice [10,12,17]. The present analysis adds a large, multi-institutional, active-comparator cohort to this literature while still requiring cautious interpretation because procedure choice was not randomized.

The absence of a statistically significant mortality difference is clinically plausible and aligns with prior MMAE studies in which recurrence or treatment failure improved more clearly than all-cause survival [9,13,18,19]. All-cause mortality after nonacute subdural hematoma reflects much more than the procedure itself: age, frailty, fall risk, anticoagulant exposure, cardiovascular disease, renal disease, baseline neurologic reserve, and competing systemic illness all influence survival [1,3,19-23]. In our matched cohort, three-month mortality exceeded 10% in both groups, which appears high if interpreted as procedure-related mortality but is more consistent with an older, comorbid, inpatient-coded real-world population and an all-cause endpoint. For this reason, the mortality findings should be framed as comparable survival after treatment selection and matching, not as evidence that one procedure is intrinsically safer than the other.

The lower incidence of newly recorded seizure diagnoses after MMAE in the primary analysis is biologically plausible but not definitive [24]. Surgical evacuation may involve cortical manipulation, irrigation, postoperative pneumocephalus, residual blood products, and perioperative physiologic stress, all of which may influence seizure risk [24]. MMAE avoids direct hematoma evacuation and cortical exposure, but it also leaves hematoma contents to resolve gradually, so a simple mechanical explanation is insufficient [15,24,25]. Importantly, the seizure endpoint in this study reflects newly recorded seizure-related diagnoses after exclusion of patients with pre-index seizure diagnoses rather than clinically or electrographically adjudicated seizures. The available data did not characterize antiseizure medication prophylaxis, detailed timing of seizure onset relative to treatment, electrographic or EEG confirmation, seizure phenotype, or relevant hematoma characteristics. These unmeasured factors may have influenced the observed between-group difference. Moreover, the seizure association became statistically nonsignificant in the sensitivity analysis restricted to patients older than 65 years. Accordingly, the observed lower incidence of coded seizure diagnoses after MMAE should be considered hypothesis-generating rather than evidence of a seizure-preventive effect and requires prospective confirmation in datasets with detailed neurological, medication, electrographic, and hematoma-level information [24,26].

Coded ischemic stroke risk was similar between groups across all follow-up intervals. This finding should be interpreted narrowly and should not be considered evidence of the overall procedural safety of MMAE. Although MMAE is minimally invasive, embolization carries procedure-specific risks, including unintended embolization through potentially hazardous anastomoses and ischemic complications involving intracranial or ophthalmic circulation [9,11,23,27,28]. The TriNetX outcomes available for this analysis did not capture the full spectrum of procedure-specific adverse events, including visual or ophthalmic complications. Moreover, administrative data do not reliably distinguish periprocedural ischemic events from delayed vascular events or preexisting neurologic deficits coded during follow-up. Accordingly, the present findings demonstrate only that coded ischemic stroke incidence did not differ significantly between the matched treatment groups and should not be interpreted as establishing comparative procedural safety.

Clinical interpretation

The comparison between standalone MMAE and surgery is clinically relevant primarily for patients in whom both strategies are realistic treatment options. Surgery remains essential when urgent decompression is required, particularly in patients with depressed consciousness, severe focal deficits, substantial mass effect, or rapid clinical deterioration [3,13,18,28]. In those scenarios, standalone MMAE should not be framed as a substitute for operative evacuation. Conversely, standalone MMAE may be considered in selected noncritical patients, patients at higher operative risk, patients with recurrent or gradually progressive hematomas, or patients in whom slower radiographic resolution is acceptable [18]. This distinction is central: the present findings should not be interpreted as establishing interchangeability between MMAE and surgery across all presentations but rather as an observational comparison of outcomes in propensity-matched treatment cohorts.

These findings also support a more nuanced role for MMAE in treatment algorithms. Surgical evacuation provides immediate relief of mass effect, while MMAE addresses the vascularized membrane biology associated with persistence and recurrence [6,9,19,22,28,29]. In practice, the choice among surgery, standalone MMAE, adjunctive MMAE, and observation should be guided by neurologic status, hematoma size and mass effect, recurrence history, anticoagulation needs, frailty, and local procedural expertise [17-19,28]. Our data are most useful for informing that risk-benefit discussion, not for replacing clinical judgment at the bedside.

Strengths and limitations

This study has several strengths. First, it uses a large multi-institutional network and an active-comparator design rather than comparing MMAE with no procedure or medical management alone. Second, the cohorts were mutually exclusive during the exposure window, which helps isolate standalone MMAE from combined or rescue strategies. Third, propensity matching included demographic factors, vascular comorbidities, type 2 diabetes mellitus, nicotine dependence, alcohol-related disorders, antiplatelet and anticoagulant exposure proxies, and available laboratory or physiologic measures. Fourth, outcomes were assessed at clinically meaningful 3-, 6-, and 12-month intervals, allowing evaluation beyond the immediate perioperative period. Finally, the analysis focuses on standalone MMAE, an area where evidence remains less mature than the adjunctive MMAE literature.

The main limitation is confounding by indication. Patients selected for surgery may have had larger or thicker hematomas, greater midline shift or mass effect, more severe neurological deficits, lower Glasgow Coma Scale scores, or a need for urgent decompression, whereas patients selected for standalone MMAE may have been more clinically stable or considered poor surgical candidates. TriNetX does not provide the radiographic and neurological detail needed to fully account for these major determinants of treatment selection. Although propensity score matching improved comparability across measured demographic and clinical characteristics, it cannot balance unmeasured severity or other unavailable determinants of treatment selection. Similarly, the critical illness proxy sensitivity analysis provided an additional assessment in a more restricted population but does not fully address or eliminate confounding by indication. Accordingly, the observed findings should be interpreted as associations within the matched cohorts rather than evidence that the treatment groups were clinically interchangeable.

Several additional limitations should be acknowledged. The diagnosis codes used to define chronic, subacute, and unspecified nontraumatic subdural hematoma may not perfectly distinguish hematoma chronicity. Moreover, the designation “nontraumatic” reflects the ICD-10-CM coding framework rather than adjudicated clinical etiology. Minor or unrecognized preceding trauma may not have been captured consistently, particularly among older adults, and some patients classified as having nontraumatic subdural hematoma may therefore have had an unrecognized traumatic contribution. Conversely, ICD-coded nontraumatic subdural hematoma represents an etiologically heterogeneous population, and the available data did not permit reliable identification or balancing of all potential underlying causes, such as specific coagulopathies, intracranial hypotension, neoplastic disease, or vascular abnormalities. These factors may have influenced both treatment selection and clinical outcomes. Hematoma subtype was not available as a separately balanced matched covariate in the output. The procedure code for MMAE identifies embolization but does not capture laterality, embolic agent, technical success, distal penetration, operator specialty, anesthesia approach, or procedural complications. Surgical codes do not distinguish burr-hole drainage, craniotomy, drain use, irrigation strategy, or postoperative management. The clinical scope of this comparison is further limited to the outcomes directly available in the TriNetX reports: all-cause mortality, ischemic stroke, and newly recorded seizure diagnoses. The database did not provide several outcomes central to treatment selection and treatment success in subdural hematoma, including hematoma recurrence, radiographic resolution or progression, repeat or rescue intervention, functional neurological outcome, and discharge disposition. It also lacks hematoma volume, membrane features, midline shift, Glasgow Coma Scale, focal deficits, modified Rankin Scale, frailty, antithrombotic restart timing, antiseizure prophylaxis, and quality-of-life outcomes. Consequently, the present study should be interpreted as a comparison of the selected coded outcomes available in these reports rather than an assessment of the overall comparative effectiveness, superiority, or equivalence of standalone MMAE versus surgical evacuation. Follow-up is based on encounters within the network, so outcome ascertainment may differ between cohorts, especially at 12 months. Finally, because proportional hazards assumptions were not met for mortality, hazard ratios should be treated as supportive rather than primary evidence.

Future directions

Future studies should directly compare standalone MMAE with surgical evacuation in carefully defined noncritical patients and should incorporate standardized imaging, neurologic severity, functional outcomes, recurrence, reintervention, antithrombotic management, and health care utilization. Randomized trials or prospective registries would be particularly valuable for identifying which patients can safely undergo standalone MMAE, which patients require immediate surgery, and which patients benefit most from a combined approach. The older adult sensitivity finding for seizures also deserves specific evaluation because age, frailty, cortical vulnerability, prophylactic medication practices, and competing mortality may alter the apparent seizure signal.

## Conclusions

In summary, standalone MMAE was associated with similar all-cause mortality and coded ischemic stroke risk compared with surgical evacuation at 3, 6, and 12 months among adults with ICD-10-CM-coded nontraumatic subdural hematoma in this propensity-matched TriNetX cohort. The primary analysis showed lower incident seizure diagnoses after MMAE, but this association was not preserved in the age >65 years sensitivity analysis and should therefore be considered hypothesis-generating. Given the potential for residual confounding by indication and the absence of detailed clinical and radiographic severity measures, these findings should not be interpreted as establishing equivalence between standalone MMAE and surgical evacuation. Further prospective comparative studies incorporating clinical and radiographic severity are warranted to better define appropriate patient selection.
